# Toward an understanding of the pathophysiology of clear cell carcinoma of the ovary (Review)

**DOI:** 10.3892/ol.2013.1550

**Published:** 2013-08-28

**Authors:** CHIHARU UEKURI, HIROSHI SHIGETOMI, SUMIRE ONO, YOSHIKAZU SASAKI, MIYUKI MATSUURA, HIROSHI KOBAYASHI

**Affiliations:** Department of Obstetrics and Gynecology, Nara Medical University, Kashihara, Nara 634-8522, Japan

**Keywords:** clear cell carcinoma, ovarian cancer, endometriosis, HNF-1β, estrogen receptor

## Abstract

Endometriosis-associated ovarian cancers demonstrate substantial morphological and genetic diversity. The transcription factor, hepatocyte nuclear factor (HNF)-1β, may be one of several key genes involved in the identity of ovarian clear cell carcinoma (CCC). The present study reviews a considerably expanded set of HNF-1β-associated genes and proteins that determine the pathophysiology of CCC. The current literature was reviewed by searching MEDLINE/PubMed. Functional interpretations of gene expression profiling in CCC are provided. Several important CCC-related genes overlap with those known to be regulated by the upregulation of HNF-1β expression, along with a lack of estrogen receptor (ER) expression. Furthermore, the genetic expression pattern in CCC resembles that of the Arias-Stella reaction, decidualization and placentation. HNF-1β regulates a subset of progesterone target genes. HNF-1β may also act as a modulator of female reproduction, playing a role in endometrial regeneration, differentiation, decidualization, glycogen synthesis, detoxification, cell cycle regulation, implantation, uterine receptivity and a successful pregnancy. In conclusion, the present study focused on reviewing the aberrant expression of CCC-specific genes and provided an update on the pathological implications and molecular functions of well-characterized CCC-specific genes.

## 1. Introduction

Epithelial ovarian cancers are a heterogeneous group with varying pathologies, and are broadly categorized into serous, mucinous, endometrioid and clear cell histotypes. These histological subtypes demonstrate morphological features that resemble the Müllerian duct-derived epithelial cells, which are embryologically derived from the fallopian tubes, endocervix, endometrium and gestational endometrium, respectively. Epithelial ovarian cancer is generally managed as a single entity, and treated with a combination of maximal cytoreductive surgery and taxane/platinum-based chemotherapy (1). Randomized clinical trials have allowed us to develop a number of successful therapeutic strategies using paclitaxel and carboplatin for serous cancer, which is the most prevalent histotype (2). However, the current standard treatments applied to high-grade serous cancer are ineffective for clear cell carcinoma (CCC) (1). CCC is more likely to be chemoresistant than high-grade serous cancer. The differential expression of chemoresistance-related genes has been observed in various histological types of ovarian cancer (1).

The intrinsic chemoresistance of CCC appears to be related to an increase in the detoxification of the drug within the cell or an increase in cell cycle arrest during the DNA damage response (3). Previous studies have demonstrated that the majority of the CCC-specific genes display a significant change in expression upon exposure to an oxidative stress microenvironment (3–5). Several studies have summarized the clinicopathological features, phenotypic and genomic changes in CCC and provided mechanistic interpretations of the gene expression changes, including chemoresistance, cell cycle regulation, hormone independency, detoxification, glycogen synthesis and chromosomal instability (3,6,7).

Endometriosis-associated neoplasms include CCC, endometrioid adenocarcinoma, serous borderline tumors, Müllerian mucinous borderline tumors, adenosarcoma and endometrial stromal sarcoma (8). Substantial histopathological data provide evidence that CCC and endometrioid adenocarcinoma are the most common histologies in ovarian cancer patients who have associated endometriosis, also known as endometriosis-associated ovarian cancer. Abundant iron-mediated oxidative stress occurs due to repeated hemorrhage in endometriosis, prior to this compound oxidatively modifying host genomic DNA stability, which is a significant factor that accelerates carcinogenesis (9). Thus, the pathogenesis of endometriosis-associated ovarian carcinogenesis may be closely associated with iron overload. Iron overload-related diseases, including hemochromatosis, chronic viral hepatitis, asbestosis and endometriosis, may lead to the development of hepatocellular carcinoma, mesothelioma or ovarian cancer (10). Such microenvironments may play a role in carcinogenesis. Nevertheless, CCC and endometrioid adenocarcinoma have distinct clinicopathological characteristics and molecular phenotypes (11,12). The present study reviews a considerably expanded functional set of CCC-specific genes and discusses recent insights into the pathophysiology of CCC.

## 2. Review of the literature

A comprehensive review of the literature was conducted in order to investigate the molecular basis of the CCC-specific genes. A MEDLINE search of the literature was performed using the key words ‘endometriosis’, ‘ovarian cancer’, ‘clear cell carcinoma’, ‘estrogen receptor’, ‘Arias-Stella reaction’ and ‘HNF-1β’. English-language publications in PubMed and references from relevant articles published between 2005 and 2012 were analyzed. References in the studies identified were also searched.

## 3. CCC-specific genetic signaling circuitries

Several genome-wide gene expression analyses have led to the identification of sets of CCC-specific genes and their regulated targets, and highlighted a complex circuitry responsible for CCC development, maintenance and progression (5). Two characteristics define CCC cells, hormone independency and HNF-1β overexpression. A previous study on the immunophenotype of CCC cells showed that the tumor cells are positive for HNF-1β and insulin-like growth factor binding protein-1 (IGFBP-1), but negative for estrogen receptor (ER), progesterone receptor (PR), Wilms tumor 1 (WT1) and p53 (13).

The expression of the hormone receptors, ER and PR, is more elevated in cells of endometrioid-type than in those of non-endometrioid origin (14). ER and PR expression may be a significant event in the progression of endometrioid carcinoma, which develops from endometrial hyperplasia with the risks of unopposed estrogen signaling in the endometriotic epithelium. Notably, CCC has shown negative or weak staining for ER-α and PR (13). The frequent loss of estrogen function may be a turning point in CCC progression and aggressiveness. DNA methylation of the promoter region, histone deacetylation, chromatin remodeling and ubiquitination may negatively regulate ER expression in CCC (11).

Among epithelial ovarian cancers, the majority of CCC cases showed the expression of the HNF-1β gene, whereas non-CCC tumors hardly expressed this gene (15). The CCC-specific genes are in extremely close linkage with HNF-1β and its target genes. HNF-1β may be a central player in the differentiation into CCC-specific lineages from endometriosis (4). There is a significant overlap between the HNF-1β-related genes and those associated with chemoresistance, detoxification and glycogen synthesis, with the conservation of the biological features of CCC (3,7,11,12). HNF-1β plays a role in the cellular stress response in CCC through its regulation of cytoprotective genes (3). Despite recent studies, our understanding of the HNF-1β-associated molecular mechanisms and functional players in CCC remains limited.

**Figure 1:**
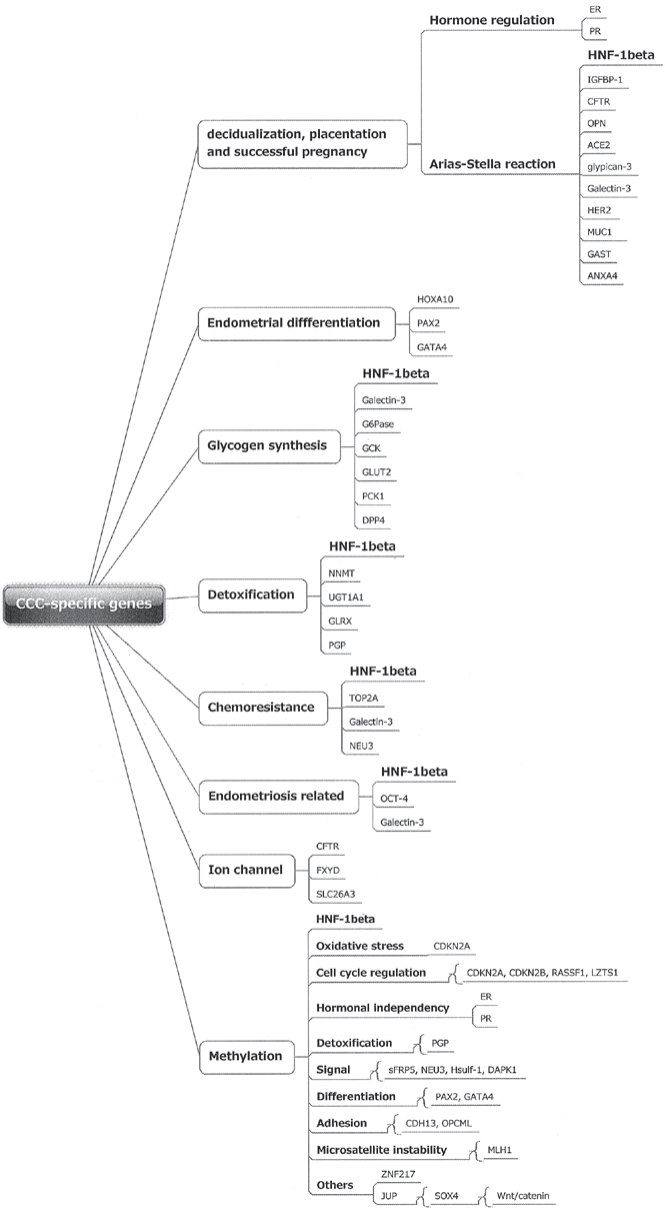
Regulation of gene expression associated with molecular functions and biological processes in clear cell carcinoma (CCC). Gene expression was analyzed with respect to genome-wide gene expression profiling and gene ontology groups. The gene tree starts with the top term ‘CCC-specific genes’ and then splits out into more specific functional terms. Each box contains the name of the process. The CCC-specific genes were classified into diverse functional categories and signalling pathways, including decidualization, endometrial differentiation, glycogen synthesis, detoxification, chemoresistance and ion channels.

To further elucidate the biological pathway network, the present study reviewed extensive functional studies of CCC-specific genes and proteins at the transcriptome and proteome levels. The CCC-specific genes were classified into several groups based on assorted physiological functions (Fig. 1). The differentially expressed genes are mainly involved in several functional groups, including those of decidualization, placentation and successful pregnancy, the Arial-Stella reaction, differentiation, glycogen synthesis, detoxification, chemoresistance, endometriosis development and ion channels. Progesterone is required to develop and maintain the decidualized phenotype of the endometrium and plays a role in epithelial-mesenchymal communication (16). The process of the differentiation of endometrial stromal cells into secretory cells, known as decidualization, is a prerequisite for the successful implantation, progression and maintenance of a pregnancy. The placenta is critical for nourishing the fetus in order to meet the requirements of the growing fetus throughout pregnancy, and also produces metabolizing enzymes that detoxify foreign chemicals. Ion channels play important roles in the feto-placental vasculature and are essential for electrolyte absorption and replacement of essential amino acids (17). These data allow us to speculate that the CCC-specific genes stimulate the secretory functions of the endometrium that are required for differentiation and implantation, placentation, and pregnancy recognition signaling, and are essential for fetal development.

### Arias-Stella reaction

Epigenetic, genetic, morphological and functional changes occurring within the endometrium during the menstrual cycle are orchestrated under the influence of sequential exposure to the cyclic expression of the ovarian steroids, estradiol and progesterone. These changes are required for the development of uterine receptivity, the disintegration of the decidualized endometrium and the subsequent repair and fine remodeling for tissue regeneration, differentiation and placentation. Two independent ERs, ER-α and ER-β, have distinctive cellular distribution patterns in the reproductive organs. ER-β predominantly inhibits the activity of ER-α. The expression of ER-α has been identified as lower during the secretory phase compared with the proliferative phase, and significantly lower in the first-trimester decidua (18). In contrast to ER-α, ER-β was observed to be expressed in all compartments of the decidual tissues. The PR gene produces two isoforms, PRA and PRB, which are mainly localized to the nuclei of stromal cells. PR was expressed at a lower level in the secretory endometrium than in the proliferative endometrium (18).

Although ER expression in endometriosis is variable, several studies have reported higher levels of ER-β and lower levels of ER-α (19). The expression of PR, particularly the PR-B isoform, is decreased, demonstrating that the resistance of endometriotic tissue to progesterone, so-called progesterone resistance, is commonly observed in females with endometriosis (19). The endometriotic ER and PR expression pattern resembles that of the eutopic secretory endometrium. Furthermore, in a study on endometrioid adenocarcinoma, ER-α expression increased with malignant transformation. By contrast, a gradual reduction in ER-α and PR expression was observed with malignant transformation from endometriosis to atypical endometriosis to CCC (20). There is a marked tendency for the disappearance of these receptors in association with the malignant transformation of endometriosis into CCC.

The Arias-Stella reaction is a hormone-induced hyperplastic change of the endometrial epithelium that is associated with cellular atypia, which may be the result of the regeneration and proliferation activities of the endometrial or endometriotic glands (21). The changes in this reaction, a common and universally recognized feature of the gestational endometrium, have been frequently observed in abortion curettage specimens (21). The reaction was also observed in the cytoplasm of stromal cells undergoing decidual change in certain patients treated with exogenous hormones, such as progestin (21). Endometriosis infrequently shows decidual changes and a Arias-Stella reaction, suggesting the possibility of a metaplastic origin from the secondary Müllerian system (22,23). Morphological and phenotypic features of the CCC cells resemble those of cells undergoing the Arias-Stella reaction (24). The Arias-Stella reaction and CCC often show prominent hobnail features and clear cell changes. The etiology for the cytoplasmic clearing observed in these cells includes the accumulation of intracellular glycogen. Phenotypical homology may emphasize common ancestry through the genetic continuity. However, ER and PR expression has shown a marked difference between endometrial epithelial cells, corresponding to the Arias-Stella reaction (positive staining), and CCC (often appears negative), indicating that clear cells in the two entities are phenotypically similar but hormonally distinct. CCC cells are able to exploit a progression mechanism via hormone-independent signaling pathways (11,21).

### Decidualization, placentation and a successful pregnancy

The unique gene families discussed in this section may be closely related to decidualization, placentation and a successful pregnancy. These proteins are expressed at a higher level in the secretory endometrium than in the proliferative endometrium. The insight provided by this review may illuminate how the hormone-independent state is established and regulated.

#### Osteopontin (OPN), also known as secreted phosphoprotein 1

OPN is a glycoprotein of the extracellular matrix that also acts as a chemokine. OPN is most likely a direct target gene of HNF-1β, as it contains functional HNF1 binding sites in its promoter region (25). OPN is expressed in the cyclic endometrium, decidual stromal cells and natural killer cells, and higher expression has been detected in the later gestational phase compared with the early gestational phase. OPN expression was shown to be regulated by progesterone (25). The involvement of OPN has been proposed for maintaining successful pregnancy. Furthermore, OPN was shown to be overexpressed in the late secretory endometrium of females with endometriosis (25). This glycoprotein has been shown to mediate cellular invasion and to contribute to tumorigenesis in several types of cancer. OPN may be involved in the pathogenesis of endometriosis and endometriosis-associated CCC, and its regulation may have a crucial role in CCC therapy (26).

#### Angiotensin converting enzyme 2 (ACE2)

The ACE2 gene contains functional HNF1 binding sites and its overexpression has been observed in CCC. ACE2 metabolizes Angiotensin II, a vasoconstrictor, to Angiotensin (1–7), a vasodilator. This enzyme stimulates endothelial cell migration, capillary formation and angiogenesis, and attenuates angiotensin II-induced reactive oxygen species (ROS) production (27). ACE2 expression has been shown to be elevated in the secretory phase of endometrial epithelial cells and early gestational trophoblasts. One of the first signs of implantation is an increase in endometrial vascular permeability and angiogenesis (27). Thus, ACE2 may act as a local autocrine/paracrine regulator of angiogenesis, vascular permeability and growth during decidualization and implantation (?).

#### Galectin-3 and lectin, galactoside-binding, soluble 3 (LGALS3)

Galectin-3 protein has been immunohistochemically observed in 59.7% of CCC cases (28). It has been implicated in cell cycle control, adhesion, migration, invasion, angiogenesis and tumor development through the activation of the Wnt signaling pathway. The overexpression of galectin-3 in CCC may contribute to cisplatin resistance (29). Galectin-3 has been shown to be expressed in endometrial epithelial cells during the secretory phase of the menstrual cycle, and to regulate cell-cell adhesion by interacting with integrin-β3 during embryo implantation (30). 17β-estradiol, progesterone and human chorionic gonadotropin induced the expression of galectin-3 (30). Galectin-3 also stimulates the expression of phosphorylated glycogen synthase kinase-3β (GSK3β) and regulates glycogen synthesis. Furthermore, galectin-3 expression has been observed to be higher in endometriosis than in the eutopic endometrium, indicating that galectin-3 has a potential role in the development of endometriosis and subsequently, in CCC (31).

#### Glypican-3 (GPC3)

The expression of GPC3, a heparan sulfate proteoglycan, has been observed in 44% of CCC cases, while rarely being observed in other histological subtypes (32). GPC3 regulates cellular growth by interacting with a variety of morphogenic or growth factors, such as Wnt, fibroblast growth factor (FGF)2 and bone morphogenic protein (BMP). The expression of GPC3 has been identified in the endometrial epithelium in the gestational period (32). The differentiated syncytiotrophoblast cells derived from the placenta expressed GPC3 mRNA and protein.

#### Mucin 1, cell surface associated (MUC1)

The cancer-associated aberrantly sialylated MUC1 glycoprotein specifically expressed on CCC plays a role in tumorigenicity, survival under anoikis conditions and malignant behavior (33). Progesterone stimulates the expression of MUC1. MUC1 has been observed to be expressed in endometriosis (34). The endometrium of fertile females also expresses MUC1, which carries selectin ligands recognized by the human blastocyst. MUC1 expression may be required at implantation sites to permit embryo attachment and implantation (34,35).

#### Annexin A4 (ANXA4)

ANXA4 expression is elevated in CCC and enhances cancer cell chemoresistance (36). This protein belongs to a family of calcium-dependent phospholipid-binding proteins and is involved in ion channel regulation, exocytosis and signal transduction. Studies have shown that ANXA4 mRNA is significantly upregulated during mid-to-late-secretory phases compared with proliferative phases, and that progesterone, but not estrogen, increased the expression of ANXA4 mRNA and protein (37). The lower expression of ANXA4 during implantation in infertile patients with endometriosis may be associated with the decrease of endometrial receptivity (38).

#### Insulin-like growth factor binding protein-1 (IGFBP-1)

IGFBP-1 is a specific marker for CCC (39). The expression of IGFBP-1 may be more specific than that of HNF-1β. The specific expression of IGFBP-1 has also been confirmed in the secretory endometrium, the decidua of the placenta and the Arias-Stella glands (39). Progesterone induces the expression of IGFBP-1. IGFBP-1 is downregulated in endometriosis via reduced levels of forkhead box O1 (FOXO1) and homeobox A10 (HOXA10), upstream target genes of IGFBP-1.

#### Human epidermal growth factor receptor 2 (HER2), also known as v-erb-b2 erythroblastic leukemia viral oncogene homolog 2

HER2/neu-positivity in CCC has been observed to be significantly higher than in other ovarian cancers (40). This oncogene showed the highest level of expression during the early secretory phase and in the placenta. HER2 plays fundamental roles in embryogenesis, development, proliferation and differentiation.

#### Octamer-binding protein 4 (OCT-4), also known as POU class 5 homeobox 1

CCC shows positive staining (28%) for the stem cell marker OCT-4, which is key in stem cell identity and reprogramming (41). The transcription factor OCT-4 is one of the most likely markers for endometrial stem cells. OCT-4 plays a role in embryonic development, particularly during early embryogenesis. Endometriotic cells express OCT-4, indicating that the ectopic endometrium in endometriosis has a stem cell origin and may explain the possible progression to ovarian cancer (42).

### Endometrial differentiation

The proteins that increase between the early and mid-luteal phases may play direct roles in embryo-uterine interactions during the implantation process (43). These implantation-related genes include HOXA10, MUC1, leukemia inhibitory factor (LIF), glycodelin, matrix metalloproteinase (MMP), tissue inhibitors of matrix metalloproteinase (TIMP) and E-cadherin. The majority of these genes are overexpressed in CCC.

#### HOXA10

In a study by Li *et al*(44) HOXA10 was not expressed in the normal ovarian epithelium, endometriosis and ovarian serous carcinomas samples, while 68.9% of the CCC samples were positive for the expression of HOXA10. HOXA10 is a DNA-binding transcription factor that regulates gene expression, morphogenesis and differentiation. This gene regulates endometrial receptivity and its expression is decreased in females with endometriosis. HOXA10 is expressed cyclically during the menstrual cycle in the endometrium, under the influence of sex steroids, with the highest expression during the implantation window (45).

### Glycogen synthesis

Glycogen accumulation operates as an energy source, enabling cell growth even under conditions of oxidative stress. Genes associated with glycogen synthesis are related to a mechanism of cisplatin (CDDP)-resistance in CCC (3,46).

#### Glucose-6-phosphatase (G6Pase)

G6Pase catalyzes the hydrolysis of D-glucose 6-phosphate to D-glucose (47). The endometrium and placenta produce glucose via G6Pase. The key metabolic change that occurs during the implantation period is the rise in endometrial glycogen content. Glycogen synthesis is regulated by progesterone (47).

#### Glucokinase (GCK), also known as hexokinase 4

This protein regulates glycerol uptake and is associated with glycogen synthesis (http://www.ncbi.nlm.nih.gov/gene/2645).

#### Glucose transporter type 2 (GLUT2)

GLUT isoforms, of which 13 have been identified to date, mediate facilitated glucose transport. GLUT2 expression increases in the endometrium throughout the secretory phase and in the decidua (48).

#### Phosphoenolpyruvate carboxykinase (PCK1)

PCK1 regulates gluconeogenesis. PCK1 forms phosphoenolpyruvate from oxaloacetate and its expression is regulated by insulin (http://www.ncbi.nlm.nih.gov/gene/5105).

#### Dipeptidyl peptidase 4 (DPP4), also known as CD26

DPP4, also known as CD26, is identical to adenosine deaminase complexing protein-2 (http://www.ncbi.nlm.nih.gov/gene/1803). DPP4 inactivates insulin-sensing incretin hormones, such as glucagon-like peptide 1 (GLP-1) and glucose-dependent insulintropic polypeptide (GIP) (49).

### Detoxification

The diagnosis of endometriosis is based on the direct or indirect evidence of cyclic bleeding in ovarian tumors. Persistent iron-induced free radicals induced by endometriosis-dependent hemorrhage may be associated with carcinogenesis. The HNF-1β-related detoxification enzymes are crucial for protection against oxidative stress. Genes associated with detoxification are also related to a mechanism of CDDP-resistance in CCC (46).

#### UDP-glycosyltransferase 1 family polypeptide A1 (UGT1A1)

UGT1A1 is an enzyme of the glucuronidation pathway that plays a critical role in the detoxification that transforms small lipophilic molecules, including estrogen, bilirubin, hormones and drugs, into water-soluble, excretable metabolites. Menstruation and hemorrhage in endometriosis are biochemically active environments known to undergo potent oxidizing reactions. Iron and bilirubin mediate the generation of ROS via the Fenton reaction. If unconjugated or uncontrolled, they may lead to oxidative DNA and protein damage. UGT1A1 is also involved in controlling the toxicity of the chemotherapeutic agent irinotecan. The presence of UGT1A1*28 may result in an increased risk of ovarian cancer (50).

#### Glutaredoxin (GLRX)

GLRX is important for the antioxidant defense and for the regulation of the cellular redox state. This protein protects cells from H_2_O_2_-induced apoptosis by regulating the redox state. GLRX plays a critical role in the detoxification of iron-induced free radicals in CCC (46).

#### Nicotinamide N-methyltransferase (NNMT)

NNMT catalyzes the *N*-methylation of nicotinamide, pyridines and other structural analogs by using *S*-adenosylmethionine as a methyl donor, playing a pivotal role in the biotransformation and detoxification of numerous xenobiotics. This gene contributes to MMP-2 expression, which induces cell invasiveness via the PI3K-AKT pathway, indicating NNMT as a novel invasion-related gene (51). This protein is most likely involved in embryo-implantation mechanisms. NNMT is also one of the genes upregulated in endometrial stromal cells in response to macrophage activation in endometriosis.

### Ion channels

The absorption of uterine cavity fluid in early pregnancy results in the closure of the lumen and allows blastocysts to make contact with the luminal epithelium (52). Fluid absorption peaks at the time of implantation through progesterone action. The expression patterns of the CCC-specific gene subsets resemble those of the ion transporting proteins involved in fluid absorption.

#### Cystic fibrosis transmembrane conductance regulator (CFTR), also known as ATP-binding cassette sub-family C, member 7 (ABCC7)

The CFTR gene encodes a member of the ATP-binding cassette (ABC) transporter superfamily. CFTR is involved in sodium and chloride absorption within the uterus. Progesterone stimulates amiloride-sensitive fluid absorption. Mutations in this gene are associated with the autosomal recessive disorder cystic fibrosis. HNF-1 interacts with multiple elements across the CFTR locus (53). CFTR appears to be overexpressed in endometriosis and CCC.

#### FXYD domain-containing ion transport regulator 2 (FXYD2)

FXYD2 is the sodium and potassium-transporting ATPase subunit γ and is expressed in the uterine endometrium. HNF-1β binding sites have been detected in the FXYD2 gene (54). Mutations in this gene have been associated with renal hypomagnesemia.

#### Solute carrier family 26, member 3 (SLC26A3)

SLC26A3 functions to transport chloride ions across the cell membrane in exchange for bicarbonate ions (55). The protein is essential for chloride absorption and is predominantly localized to the mucosa of the lower intestinal tract.

### Cell cycle regulation

Genes mapping to amplified regions in CCC include 20q13.2 (harboring ZNF217), 17q12-q21.32 (harboring HER2, TOP2A, GAST, JUP and BRCA1) and 17q23.2 (PPM1D) (40). The resistance of CCC to platinum-based chemotherapy may be caused by low levels of cell proliferation (6).

#### Early mitotic inhibitor-1 (Emi1)

Significant overexpression of Emi1 protein was present in 82% of CCC (55,56). Emi1 protein is a key cell cycle regulator that promotes S-phase and mitotic entry by inhibiting the anaphase promoting complex. The overexpression of Emi1 leads to tetraploidy and genomic instability.

#### Zinc finger protein 217 (ZNF217)

ZNF217 amplification is observed in 36% of CCC, but rarely detected in serous cancer, regardless of grade (57). ZNF217 plays a role in the chemoresistance and poor prognosis in breast and ovarian cancer, possibly through the Aurora-A signaling cascade.

#### Topoisomerase (DNA) II α 170kDa (TOP2A)

TOP2A is a cell cycle regulating gene that is upregulated in chemoresistant ovarian cancer, particularly in CCC (58). TOP2 is involved in processes, including chromosome condensation, chromatid separation and the relief of torsional stress that occurs during DNA transcription and replication.

Together, all these data indicate that the CCC-specific genes significantly overlap with the genes associated with the conservation of the biological features of CCC.

## 4. CCC-specific epigenetic signaling circuitries

The changes in the DNA hypermethylation pattern is one of the mechanisms for the earliest molecular changes in carcinogenesis. In epithelial ovarian cancer, the frequently methylated genes were reported to be the cyclin-dependent kinase inhibitor 2B (CDKN2B); estrogen receptor 1 (ESR1); secreted frizzled-related protein 5 (sFRP5); cadherin 13 (CDH13, H-cadherin); Ras association (RalGDS/AF-6) domain family member 1 (RASSF1); mutL homolog 1, colon cancer, nonpolyposis type 2 (MLH1); opioid binding protein/cell adhesion molecule-like (OPCML); sulfatase 1 (Hsulf-1); GATA binding protein 4 (GATA4) and death-associated protein kinase 1 (DAPK1) genes (59,60). CCC samples exhibited a higher frequency of CDKN2B, ESR1 and sFRP5 promoter hypermethylation compared with those of other histological types (Fig. 1). In addition, homozygous deletions were detected at the CDKN2A, CDKN2B and LZTS1 loci in CCC (57). Genetic events in CDKN2A and ESR1 genes are rare in endometriosis.

### Cell cycle regulation

#### CDKN2B, also known as p15INK4B, p15, inhibits cyclin-dependent kinase 4, and CDKN2A, also known as P16INK4A

The iron-mediated formation of free radicals (e.g. ROS) has been shown to be associated with carcinogenesis. Akatsuka *et al* identified CDKN2A, a cell cycle control gene, and CDKN2B genes in a ferric nitrilotriacetate-induced renal carcinogenesis animal model (61). The two genes may be vulnerable to ROS in endometriosis. The allelic loss of the CDKN2A gene occurs specifically at the p16 loci.

#### RASSF1

RASSF1 is commonly silenced by promoter hypermethylation in a variety of types of human cancer, including ovarian cancer (62). This protein interacts with the DNA repair protein, xeroderma pigmentosum, complementation group A (XPA) and inhibits the accumulation of cyclin D1, thus inducing cell cycle arrest. No epigenetic alterations were identified in RASSF1 in endometriosis samples (63).

#### Leucine zipper, putative tumor suppressor 1 (LZTS1)

Fasciculation and elongation protein-ζ1 (FEZ1) expression is absent or markedly reduced in 38% of ovarian cancers. Homozygous deletions are detected at the LZTS1 loci at 8p22 in CCC. LZTS1 has a role in cell-cycle control by interacting with the Cdk1-cyclin B1 complex (57).

### Hormonal regulation

#### ESR1

The hormonal receptor profile of CCC and endometriosis is characterized by the low expression of ER-α and PR, and by ER-β overexpression (64). Hypomethylation at the ER-β promoter is responsible for high ER-β levels (19). ER-β suppresses ER-α levels. An increased ER-β to ER-α ratio is responsible for decreased PR expression.

### Detoxification

#### P-glycoprotein (PGP)

CCC has a lower expression of multi-drug resistance PGP than serous cancer in females (65). The proliferative endometrium has revealed no PGP expression, while the secretory and menstrual endometrium has been identified with positive staining. Progesterone increases PGP expression and function. All gestational endometria have shown positive staining for PGP in the Arias-Stella reaction and the decidua. PGP protects the fetus from exposure to xenobiotics during pregnancy (66).

### Signaling

#### sFRP5

The sFRP5 promoter has been shown to be predominantly methylated in CCC tissues, with 64.6% in CCC compared with 13.3% in serous cancer, and 0% in endometriosis and the normal ovarian epithelium (60). sFRP5 modulates Wnt signals, which play a significant role in reproductive events. sFRP5 may regulate endometrial stromal cell proliferation, survival and differentiation, which is required to support the developing embryo.

#### NEU3 (encodes sialidase 3)

Plasma membrane-associated NEU3 is expressed in the majority of CCC cases. The overexpression of NEU3 significantly enhances cell resistance to hypoxia (67).

#### Hsulf-1

Heparan sulfate 6-O-endosulfatases, such as HSulf-1, selectively remove 6-O-sulfate from heparan sulfate, upregulate heparin-binding growth factor signaling and confer resistance to chemotherapy-induced apoptosis. HSulf-1 inactivation in CCC is partly mediated by the loss of heterozygosity and epigenetic silencing (68). HNF-1β negatively regulates HSulf-1 expression.

#### DAPK1

DAPK1 is a positive mediator of TNF-α and γ-interferon-induced apoptosis via the NF-κB signaling pathways. Collins *et al* reported low levels of DAPK1 expression in CCC compared with in normal samples (69).

### Differentiation

#### Paired-box gene 2 (PAX2)

PAX2 is a target of transcriptional suppression by the tumor suppressor gene, WT1, and is essential in embryonic development of Müllerian organs. Promoter hypomethylation of the transcription factor PAX2 has been identified in 75% of CCC cases (70).

#### GATA4

The family of zinc finger-containing GATA transcription factors is frequently lost in ovarian cancer, and this loss accounts for the dedifferentiation of the cancer cells (71). GATA4 has also been shown to be frequently lost in preneoplastic lesions, including morphologically normal inclusion cysts, epithelial hyperplasia or atypical endometriosis adjacent to malignant cells. GATA4 plays critical roles in cell lineage specification during early embryonic development and organ formation.

### Adhesion

#### CDH13

CDH13, glutathione S-transferase-π1 (GSTP1) and RASSF1 are frequently methylated in sporadic and BRCA1-associated ovarian cancers. CDH13 is a cell adherence protein and is often silenced in cancer cells. Like CCC, epigenetic alterations in CDH13 and CDKN2A have been observed in a silica-induced lung cancer model (72).

#### OPCML

OPCML is frequently inactivated by allelic loss and CpG island promoter methylation in epithelial ovarian cancer (73). OPCML may have an accessory role in opioid receptor function and negatively regulate a specific repertoire of receptor tyrosine kinases, including EPH receptor A2 (EPHA2), fibroblast growth factor receptor 1 (FGFR1), FGFR3, HER2 and HER4.

### Microsatellite instability

#### MLH1

Microsatellite instability is proposed to be limited to CCC and endometrioid cancer. The epigenetic inactivation of hMLH1 is also an early event in the malignant transformation of endometriosis (74). Abnormal methylation has been detected in ~10% of endometriosis cases.

## 5. HNF-1β and its related genes

Members of the HNF-1 protein family, HNF-1α and HNF-1β, are homeobox transcription factors involved in embryonic development and tissue-specific gene expression in several organs, including the ovaries and uterus. HNF-1β plays a crucial role in cell differentiation and tissue morphogenesis. HNF-1β-knockout is embryonic lethal due to the defective differentiation of the extra-embryonic visceral endoderm (75). This transcription factor controls endoderm development. HNF-1β may be activated during the differentiation of embryonic endodermal stem cells.

In the kidney, HNF-1β is upregulated after acute kidney injury in proximal tubular cells. HNF-1β mutations have been associated with a variety of disorders of renal development with polycystic kidney disease (76). HNF-1β controls cellular proliferation and tubule formation by regulating the expression of a number of kidney-specific genes, including polycystic kidney and hepatic disease 1 (PKHD1), uromodulin (UMOD) and suppressor of cytokine signaling-3 (SOCS3) expression and the signal transducer and activator of transcription 3 (STAT3)/mitogen-activated protein kinase 1 (MAPK1, Erk) activation cascade (77).

The intestinal epithelium is a complex system characterized by continuous cell renewal, differentiation and apoptosis. In the gut, the clusters of co-regulated genes associated with HNF-1β are HNF-1α, HNF-4α, FABP1 and UGT2B7 (78). HNF-1β is also able to control the expression of a number of intestinal target genes, including FABP1, LPH, CFTR, G6Pase and DPP4. The concerted action of HNF-1α and HNF-1β activates the expression of Notch, SLC26A3, ATOH1 and JAG1, which act on cell fate determination, stem cell self-renewal, epithelial cell polarity, adhesion, cell division, differentiation and intestinal water absorption.

In the uterine endometrium, epithelial glandular nuclei have demonstrated no HNF-1β expression in the proliferative phase, with significant increases in the secretory and menstrual phases (46). The pregnant endometrial glandular cells have also been demonstrated to be uniformly and continuously positive for HNF-1β. The majority of HNF-1β-associated gene products co-localize to the cilium, a crucial organelle that plays a significant role in controlling proliferation and differentiation (79). The present review identifies direct and indirect target genes of HNF-1β and shows that this transcription factor plays a crucial role in defining cell fate and controlling terminal functions in the endoderm epithelium.

### Polycystic kidney and hepatic disease 1 (PKHD1)

This gene modulates calcium-dependent renal epithelial cell proliferation and differentiation. PKHD1 mutations cause autosomal recessive polycystic kidney disease (80).

### Uromodulin (UMOD)

UMOD in urine provides a defense against urinary tract infections (81). UMOD also acts as a constitutive inhibitor of calcium crystallization.

### Suppressor of cytokine signaling 3 (SOCS3)

SOCS3 has emerged as a critical attenuator of cytokine-mediated processes in a negative-feedback mechanism to hinder cytokine receptor activity, indicating a role in the suppression of tumorigenesis. The DNA-hypermethylation of SOCS genes in ovarian cancer has led to speculation that silencing of the SOCS3 gene may promote the oncogenic transformation of epithelial tissues (82). In the human endometrium, stromal cell decidualization has been induced in response to the expression of SOCS3, which was regulated by hormonal stimulation (83).

### Jagged 1 (JAG1)

JAG1 is the ligand for the receptor Notch1 and is involved in the biological processes of cell adhesion, motility, cell cycle regulation, cell communication and angiogenesis. Notch1 is expressed in endometrial epithelial and stromal cells, and mediates stromal differentiation and decidualization. Notch1 and 2 are believed to be stem cell markers for ovarian cancer (84).

The present review shows that HNF-1β is involved in the differentiation program of tissue structures and tissue-specific lineage in several organs, including the ovaries, endometrium, liver, pancreas, kidneys and intestine.

## 6. Summary

CCC has several significant characteristics, based on morphological, behavioral and molecular features, which are distinct from those of other ovarian cancer histologies (85). CCC is the most common entity in Japan, accounting for ~70% of all endometriosis-associated ovarian cancers. The scope of computational technologies, including DNA microarrays, genome-wide gene expression profiling, microRNAs, methylation arrays and subsequent methods for the visualization of these datasets, has been extended to various transcriptomic and proteomic features that are focused on individual platforms (86).

The present review systematically identified and reevaluated the CCC-associated genes (Fig. 1). Firstly, due to advancement in computational predictions, HNF-1β has been unveiled as a major hub in the biology of CCC (91). With promoter hypomethylation, the expression of HNF-1β is significantly upregulated in endometriosis and endometriosis-associated ovarian CCC. HNF-1β is believed to be a master regulator of endodermal organogenesis and has long been discussed with regard to its role in clear cell carcinogenesis (3–5,12,15,46,56,85–87). While the overexpression of HNF-1β may lead to cell regeneration, its potential role in malignant transformation has remained obscure. The present findings of this review highlight the importance of the HNF-1β-induced global reproductive gene expression changes on morphology and ultimately on tissue/organ function. These changes included the process of the abnormal gain or loss of several significant genes, including decidualization, endometrial differentiation and regeneration, hormonal dependency, glycogen synthesis, detoxification, ion exchange and cell cycle regulation (3,4,12,46,56,85). Collectively, the present review uncovers an unanticipated link between HNF-1β upregulation in CCC and the acquisition of cell cycle regulation under conditions of oxidative stress and inflammation. Cell cycle arrest may be a failsafe mechanism against the oxidative stress-induced DNA damage response (88).

Secondly, the present review demonstrated that HNF-1β is a strong inducer of endometrial receptivity, a morphogenetic program that is key to successful pregnancy. The endometrium undergoes morphological and functional changes during the menstrual cycle, which are essential for uterine receptivity (12,56,88). These changes are driven by estrogen and progesterone and involve the fine control of numerous different genes, which have been induced by epigenetically-regulated HNF-1β (87). The specific expression of HNF-1β and its target genes has been confirmed in not only endometriosis and CCC, but also the secretory endometrium, the Arias-Stella glands and the decidua of the placenta (11). The Arias-Stella reaction may be the result of the regeneration and proliferation activity of the endometrial glands. A number of the HNF-1β target proteins may be hormonally-regulated and involved in the uteroplacental transport of substrates important in the implantation process and in early embryo-endometrial interactions (46,56). The ectopic overexpression of HNF-1β in the normal secretory and gestational endometria may be sufficient to induce the morphological and molecular changes characteristic of a successful pregnancy. HNF-1β may be an activator to establish female reproduction, including secretory phase organogenesis, endometrial regeneration, differentiation, decidualization, glycogen synthesis, detoxification, cell cycle regulation, implantation, uterine receptivity, placentation and a successful pregnancy.

Thirdly, the overexpression of HNF-1β along with a lack of ER/PR expression may be used as a sensitive model system to identify the molecular characteristics of CCC. ER-α/ER-β and PRA/PRB are frequently expressed in ovarian cancer with a certain variability relating to histological subtype, grade and stage. In total, 70–100% of serous, mucinous and endometrioid cancers show positive nuclear staining for ER-α. Conversely, CCC is negative for ER-α (89). ER-β staining for CCC is similar to that of non-CCC. PRs have been detected in only 10% of CCC cases compared with non-CCC (80–90%). DNA methylation and chromatin remodeling are two epigenetic mechanisms that have been linked with the lack of ER-α expression (90). These epigenetically modified genes may be involved in the expression of HNF-1β and hormone receptors.

Finally, cellular glycogen accumulation due to the promotion of glycogen synthesis is the most conspicuous feature of CCC (91). The regulated expression of the CCC-specific genes offers a mechanism to control the glycogen accumulation processes. HNF-1β regulates numerous aspects of glycogen synthesis and its target genes also delineate their interactions with signaling pathways in dictating glycogen synthesis. Glycogen is synthesized from glucose presumably to act as a source of energy for CCC cells and endometrial cells at implantation, placentation and successful pregnancy.

The present review expanded the repertoire of HNF-1β target genes, its downstream targets and its associated biological consequences, thereby shedding light on the complex regulatory circuitries of CCC. The evolutionarily conserved HNF family of transcription factors may play fundamental roles in regulating the hormonal microenvironment, secretory endometrial cell specification, glycogen synthesis and detoxification during pregnancy. The characteristics of two different entities, CCC cells and gestational endometrial cells (e.g. the Arias-Stella reaction), exhibit certain shared phenotypic and genetic features.

In conclusion, the results of the present review provide support for a marked degree of genetic overlap between CCC carcinogenesis and early gestational development.
